# Ruvidar^®^—An Effective Anti-Herpes Simplex Virus Agent

**DOI:** 10.3390/v17091280

**Published:** 2025-09-20

**Authors:** Kevin M. Coombs, Roger DuMoulin-White, Arkady Mandel

**Affiliations:** 1 Department of Medical Microbiology and Infectious Diseases, University of Manitoba, Winnipeg, MB R3E 0J9, Canada; 2 Theralase^®^ Technologies Inc., 41 Hollinger Road, Toronto, ON M4B 3G4, Canada; rwhite@theralase.com (R.D.-W.); amandel@theralase.com (A.M.)

**Keywords:** antivirals, lipid envelope, photodynamic therapy, Ruvidar^®^, Rutherrin^®^, TLD-1433, acyclovir, metformin

## Abstract

Infectious agents account for millions of deaths every year. The Herpes Simplex Viruses (HSVs) are large double-stranded DNA viruses that infect more than 90% of the human population and can establish life-long latency in human hosts. Currently, effective FDA approved anti-herpetic drugs include acyclovir and later-generation derivatives (valacyclovir and famciclovir), which inhibit viral DNA synthesis. In previous work, we demonstrated that the small molecule Ruvidar^®^ could inhibit numerous pathogenic human viruses when added to solutions of viruses both with and without light activation. In these experiments, we evaluated the ability of Ruvidar^®^ to restrict HSV-1 replication in Vero cells, both by itself and in combination with acyclovir and metformin in the absence of light activation to mimic deep tissue. Ruvidar^®^ successfully inhibited HSV-1 replication at significantly lower concentrations and more effectively than either acyclovir or metformin alone. We also discovered additive and synergistic anti-HSV-1 effects when combinational therapy was tested. Ruvidar^®^ also restricted HSV-1 replication in human U251 glioblastoma astrocytoma cells, remained highly effective against acyclovir-resistant HSV-1 mutants, and protected infected cells from virus-induced cytopathology.

## 1. Introduction

Infectious agents account for millions of deaths every year (www.who.int/data/gho/data/themes/mortality-and-global-health-estimates/ghe-leading-causes-of-death, accessed on 8 January 2025). Currently, the most effective ways to protect against infection involve the use of vaccines and anti-microbials. Vaccines are useful when administered prior to infection to allow immunity to develop, whereas antibiotics and antivirals are most useful before immunity to a vaccine has had time to develop. The primary disadvantages of vaccines are that knowledge of the agent is required in advance to manufacture an effective vaccine, and substantial time is needed to produce relevant vaccines. Furthermore, the developed vaccine may not match the eventual strain that circulates [1,2]. A growing number of anti-viral agents have been developed and some are effective against numerous viruses; however, because viruses replicate and many lack genome proof-reading capabilities, resistance to the anti-viral agent may develop rapidly [3,4,5].

The herpesviruses are large double-stranded DNA viruses (reviewed in [6]). Several different types, including Herpes Simplex Virus (HSV), type 1 (HSV-1), HSV-2, Cytomegalovirus, Varicella Zoster virus (chickenpox), and Kaposi’s sarcoma human herpes virus, have been described in the literature. In addition to causing acute infections, human herpesviruses have the capacity to establish life-long latency. Since many different herpesviruses—many of which are capable of infecting more than 90% of the population—exist, they are a significant human health concern [7]. Several anti-herpetic drugs have been developed. The “gold standard” is acyclovir. Acyclovir is a nucleoside analog that inhibits viral DNA synthesis. Due to the risk of developing resistance, several derivatives, such as valacyclovir and famciclovir, have been developed. A variety of other agents, such as foscarnet and adefovir (reviewed in [8,9,10]), also are considered effective anti-herpetic compounds.

We previously demonstrated that the small molecule Ruvidar^®^ could inhibit numerous pathogenic human viruses, when added to solutions of viruses, both with and without light activation [11]. When light-activated with green light, 2.4 nM Ruvidar^®^ was able to inhibit 50% of HSV-1 infectivity, while 53 nM was able to inhibit 99.9% of HSV-1 infectivity. Even if not light-activated, 40 nM and 200 nM Ruvidar^®^ were able to inhibit 50% and 99.9% HSV-1 infectivity, respectively.

Ruvidar^®^ can be activated by several different methodologies. As a light-activated small molecule, the primary method involves activation by an appropriate wavelength and energy of light. In the case of Ruvidar^®^, 45 Joules per cm^2^ of green light (wavelength 520 nm) is sufficient to induce photoactivation and the subsequent generation of Reactive Oxygen Species (ROS), specifically singlet oxygen, which has been demonstrated to modify lipids [11]. Secondarily, Ruvidar^®^ can be activated by various forms of ionized radiation, and thirdly by the use of ROS-producing drugs, such as the popular diabetes drug metformin (https://theralase.com/theralase-demonstrates-unique-ability-to-activate-rutherrin-with-diabetes-drug/, accessed on 21 February 2025). Metformin is an FDA drug approved for the treatment of type 2 diabetes [12,13,14] that affects major metabolic pathways, including AMPK and mTORC1 [15], and amino acid homeostasis [16]. Thus, in some clinical settings where light may not be able to penetrate, it would be advantageous to use an alternate activation method, such as metformin, which is also able to reduce HSV-1 replication on its own [17].

In our latest research, we evaluated the ability of Ruvidar^®^ to restrict HSV-1 replication in Vero cells and in human U251 glioblastoma astrocytoma cells in the absence of light activation. Ruvidar^®^ appeared to be more effective than acyclovir, both when added pre-infection (prophylactically) and post-infection (therapeutically). When added prophylactically, 10 µM Ruvidar^®^ inhibited HSV-1 replication 99.993% in both Vero cells and in U251 cells, whereas 20 µM acyclovir inhibited HSV-1 replication 99.88% in Vero cells. When used therapeutically, 24 h post-infection, acyclovir had no effect on HSV-1 replication; however, 10 µM Ruvidar^®^ was able to inhibit HSV-1 replication >99.99% in both cell types. When acyclovir and Ruvidar^®^ were used in combination in Vero cells, there was an additive anti-HSV-1 effect. Furthermore, the combination was synergistic when used prophylactically. Combinations of Ruvidar^®^ and metformin demonstrated additive effects in most cases.

## 2. Materials and Methods

### 2.1. Cells and Viruses

Monkey kidney Vero cells (ATCC #CCL-81) were cultured in DMEM supplemented with 1× L-glutamine, 1× non-essential amino acids, 1× sodium pyruvate, and 10% FBS (Gibco; Ottawa, ON, Canada, Cat #10437028). Human U-251 glioblastoma astrocytoma [U-251 MG (formerly known as U-373 MG; European Collection of Authenticated Cell Cultures—ECACC 09063001)] were cultured in DMEM/F12 supplemented with 1× L-glutamine, 1× non-essential amino acids, 1× sodium pyruvate, and 10% FBS. Cells were trypsinized and sub-cultured at 1:6–1:10 ratios 3 times a week. HSV-1 (strain F) was propagated and titrated in Vero cells.

### 2.2. Drug Treatments

Ruvidar^®^ was freshly dissolved in sterile d.H_2_O and the concentration was determined by OD_440_ as described in [11]. Acyclovir was dissolved to 100 mM in sterile DMSO. Metformin was dissolved to 2 M in d.H_2_O. Working 100× sub-stocks were prepared such that the final DMSO concentration did not exceed 0.5%. Vero and U251 cells were treated with various concentrations of each of the compounds at various times pre- and post HSV-1 infection.

### 2.3. Cell Viability Determinations

Sets of Vero cells and U251 cells in 96-well plates (5 replicates) were treated with various concentrations of each drug for 72 h and cell viabilities determined by the WST-1 reagent according to the manufacturer’s directions. Absorbances and cell viabilities were compared to non-drug-treated and to media-only blank controls, and, because Ruvidar^®^ is a deep red color, to Ruvidar^®^-in-media blank controls.

### 2.4. Selection of Drug-Resistant HSV-1 Mutants

HSV-1-infected Vero cells were treated through seven passages with increasing doses of drugs. For generation of acyclovir-resistant HSV (HSV^Ac-R^), cells were treated with 1.25 μM acyclovir for two passages, in the additional presence of 1.0 μg/mL 5-bromodeoxyuridine (BUdr) during the initial passage to enhance mutational effects [18]. Resultant supernatants were then treated with 3.16 μM acyclovir for two passages, and plaque-purified in agarose containing 10 μM acyclovir. Ten individual well-separated plaques were then amplified through two final passages in the presence of 10 μM acyclovir. The sensitivities of resultant clones were then tested against 10.0 and 20.0 μM acyclovir.

To attempt to create Ruvidar^®^-resistant HSV clones (HSV^Ru-R^), we followed a similar strategy, using 1.25, 3.16, and 10 μM Ruvidar^®^, but for two, three, and four passages, respectively, for a total of nine passages. Sensitivities of resultant clones were then tested against 10.0 μM Ruvidar^®^.

### 2.5. Infectious Virus Enumeration

Virus yields from treated cells were determined by plating 1:10 dilutions of each sample on 12-well plates of Vero cells. Cells were overlaid with 0.6% Agarose in 1× M199 supplemented with 3% FBS and 1 × L-glutamine, incubated at 37 °C for 4 days, and stained with 0.04% neutral red. Virus yields were compared to yields from non-treated controls.

## 3. Results

### 3.1. Ruvidar^®^, Acyclovir, and Metformin All Demonstrate Anti-HSV-1 Activity When Used Prophylactically

**Figure 1:**
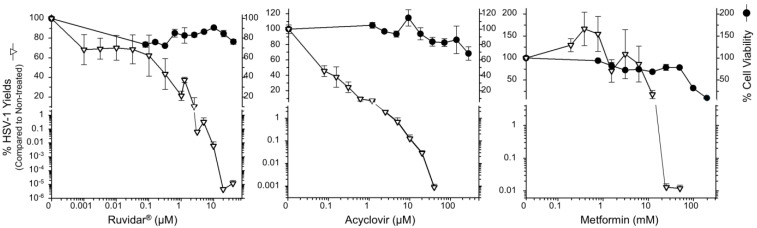
The effects of Ruvidar^®^, acyclovir, and metformin on Vero cell viability and HSV-1 yields. Vero cells were treated with the indicated concentrations of drugs for 72 h to measure cell viability, or cells were pre-treated for 4 h with the indicated drug concentrations, infected with HSV-1 at MOI ~0.01, incubated in the presence of the same drug concentrations as pre-treatment for 68 h, and virus yields were determined. Error bars represent the standard error of the means (SEMs) from at least three replicates.

We initially tested each drug’s toxicity and their capacity to inhibit HSV-1 replication in Vero cells when each drug was added to cells 4 h prior to infection and the same concentrations of drug were maintained throughout infection in the absence of light activation (Figure 1).

Cell viability remained above 60% at all tested Ruvidar^®^ and acyclovir concentrations, so a Selectivity Index (SI) could not be determined for these drugs. Metformin’s SI was calculated as 8.4; 50% cell viability was observed at 76 mM and 50% virus inhibition at 9 mM. Acyclovir was highly effective as an anti-herpetic compound, as demonstrated in numerous previous studies (i.e., [19,20,21]). Additionally, 10 µM acyclovir reduced HSV-1 replication by approximately 3 Log_10_, to 0.12% that of the untreated sample, and 40 µM acyclovir reduced HSV-1 replication by approximately 5 Log_10_, to ~8.3 × 10^−4^% of the untreated sample.

Ruvidar^®^ was more effective: 3 µM reduced HSV-1 replication to 0.06%; 10 µM reduced HSV-1 replication to ~6.5 × 10^−3^%; and 20 µM Ruvidar^®^ reduced HSV-1 replication approximately 7.5 Log_10_, to <10^−5^% of the untreated sample. Metformin by itself also showed anti-HSV-1 activity; 12.5 mM inhibited ~80% HSV-1 replication; and 25 mM metformin reduced HSV-1 replication by almost 4 Log_10_ to ~0.012% of the untreated sample. These results indicate that all three drugs are effective when used prophylactically.

### 3.2. Ruvidar^®^ Alone Demonstrates Anti-HSV-1 Activity When Used Therapeutically

**Figure 2:**
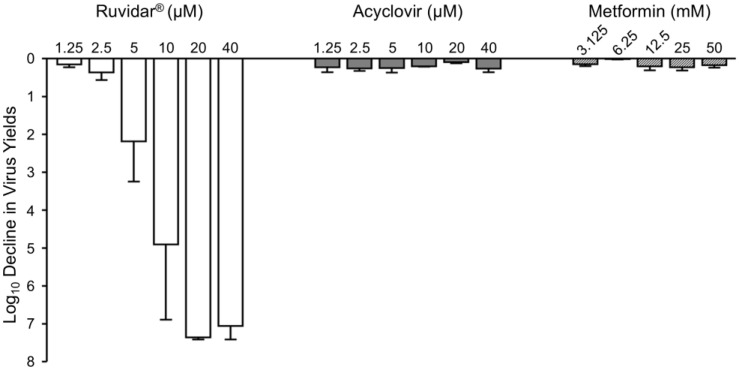
Effects of Ruvidar^®^, acyclovir, and metformin on HSV-1 yields when added 24 hpi. Vero cells were infected with HSV-1 at MOI ~ 1.5, incubated for 24 h, then treated at 24 hpi with the indicated concentrations of drugs for an additional 44 h. Virus yields were then determined, and reductions in virus yields were compared to non-treated controls. Error bars represent SEMs from at least three replicates.

We then examined each drug’s effectiveness, if used therapeutically (added 24 h after HSV-1 infection had been established) (Figure 2).

Ruvidar^®^ continued to dramatically inhibit HSV-1 replication, even when added after HSV-1 infection had been established. It was found that 5 µM Ruvidar^®^ inhibited HSV-1 replication more than 2 Log_10_, 10 µM Ruvidar^®^ inhibited HSV-1 replication approximately 5 Log_10_, and 20 µM Ruvidar^®^ inhibited HSV-1 replication approximately 7.3 Log_10_. Acyclovir had no major impact on HSV-1 replication at any tested concentration, if added 24 h post-infection (hpi), consistent with its known mode of action on viral DNA synthesis, which occurs relatively early in viral replication. Similarly, none of the tested metformin concentrations had a major impact on HSV-1 replication if added 24 hpi.

**Figure 3:**
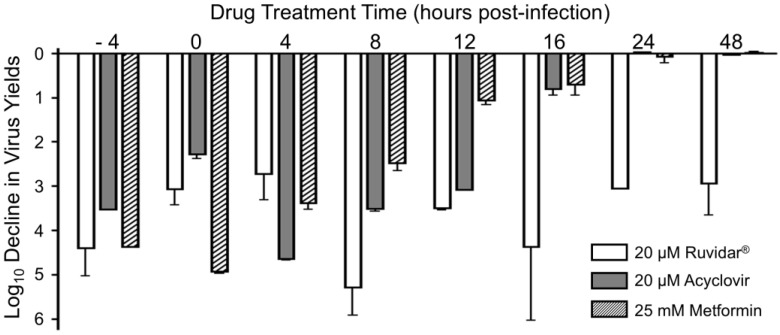
Effects of Ruvidar^®^, acyclovir, and metformin on HSV-1 yields when added at various times pre- and post-infection. Vero cells were infected with HSV-1 at MOI ~ 1.5 and treated at indicated times with the indicated concentrations of drugs. Virus yields were determined at 68 hpi, and reductions in virus yields were compared to non-treated controls. Error bars represent SEMs from at least three replicates.

To determine the kinetics of each drug’s inhibitory effects, we treated Vero cells with 20 µM Ruvidar^®^, 20 µM acyclovir, or 25 mM metformin at various times pre- and post-infection (Figure 3). There were gradual changes in acyclovir’s anti-HSV-1 activity after 4 hpi and a dramatic change (~2 Log_10_) in acyclovir’s capacity to inhibit HSV-1 replication between 12 hpi and 16 hpi, consistent with its known mode of action on viral DNA synthesis. Metformin appeared to have earlier anti-HSV-1 effects; there were gradual changes in metformin’s anti-HSV-1 activity after 0 hpi and metformin had a more dramatic effect on HSV-1 replication at 12 hpi than acyclovir. Both drugs had a <1 Log_10_ effect on HSV-1 replication by 16 hpi. By contrast, Ruvidar^®^ continued to inhibit HSV-1 replication by almost 99.9%, even when added 48 hpi.

### 3.3. Ruvidar^®^ and Acyclovir in Combination Had a Greater Anti-HSV-1 Effect

**Figure 4:**
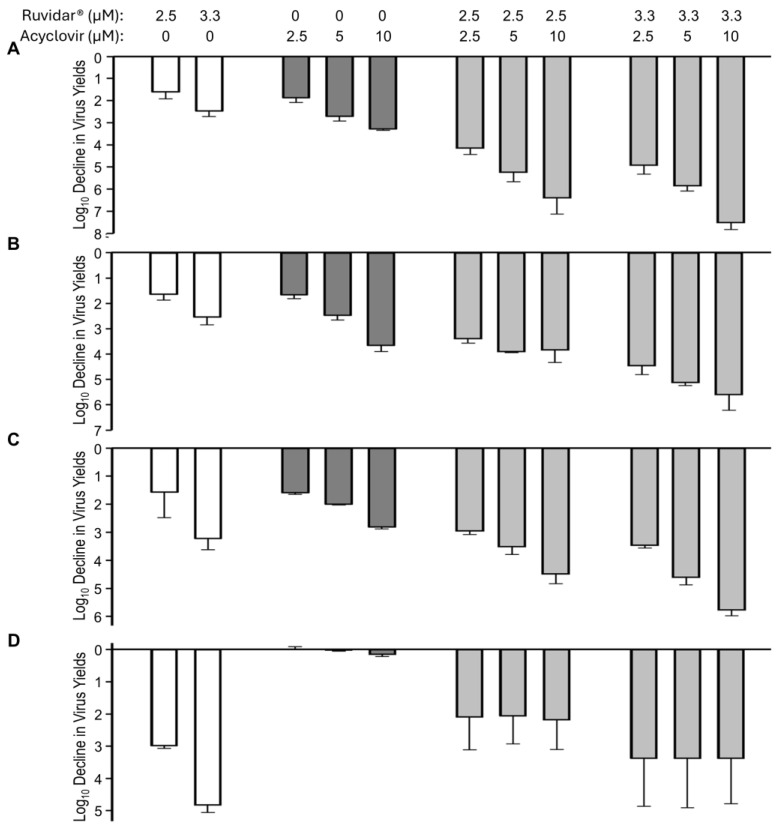
The effects of Ruvidar^®^ in combination with acyclovir on HSV-1 yields when added at various times pre- and post-infection. Vero cells were infected with HSV-1 at MOI ~ 1.5 and treated at (**A**) 4 h pre-infection; (**B**) at the same time as infection; (**C**) 8 hpi; or (**D**) 24 hpi with indicated concentrations of drugs. Virus yields were determined at 68 hpi, and reductions in virus yields were compared to non-treated controls. Error bars represent SEM from at least three replicates.

Many therapeutic drugs are more effective when used as a combinational therapy. For example, human immunodeficiency virus type 1 (HSV-1) is clinically inhibited by Highly Active Anti-Retroviral Therapy (HAART), which uses a combination of two reverse transcriptase inhibitors and either another non-nucleoside reverse transcriptase inhibitor or a protease inhibitor (reviewed in [22,23,24]). We tested various combinations of Ruvidar^®^ with either various concentrations of acyclovir or metformin, both prophylactically and therapeutically. Combinations of Ruvidar^®^ and acyclovir are shown in Figure 4.

When used alone and when added 4 h pre-infection (prophylactically), 2.5 and 3.3 µM Ruvidar^®^ inhibited HSV-1 replication by ~1.5 and ~2.5 Log_10_, respectively (Figure 4A, left-most white bars). Similarly, when used alone, and when added 4 h pre-infection, 2.5, 5 and 10 µM acyclovir inhibited HSV-1 replication ~1.8, ~2.6, and ~3 Log_10_, respectively (Figure 4A, dark gray bars). When used in combination, most doses had an additive effect (Figure 4A, right-most light gray bars); however, 3.3 µM Ruvidar^®^ combined with 10 µM acyclovir had a synergistic effect (*p* = 0.015), inhibiting HSV-1 replication by approximately 7.5 Log_10_.

The combination of Ruvidar^®^ with acyclovir had additive inhibitory effects on HSV-1 replication when added at the same time infection was initiated (Figure 4B) or if drugs were added 8 hpi (Figure 4C); however, if combinational drugs were added 24 hpi, HSV-1 replication inhibition appeared to be slightly less effective than if Ruvidar^®^ was used alone at this time (Figure 4D), although there was greater experimental variability.

**Figure 5:**
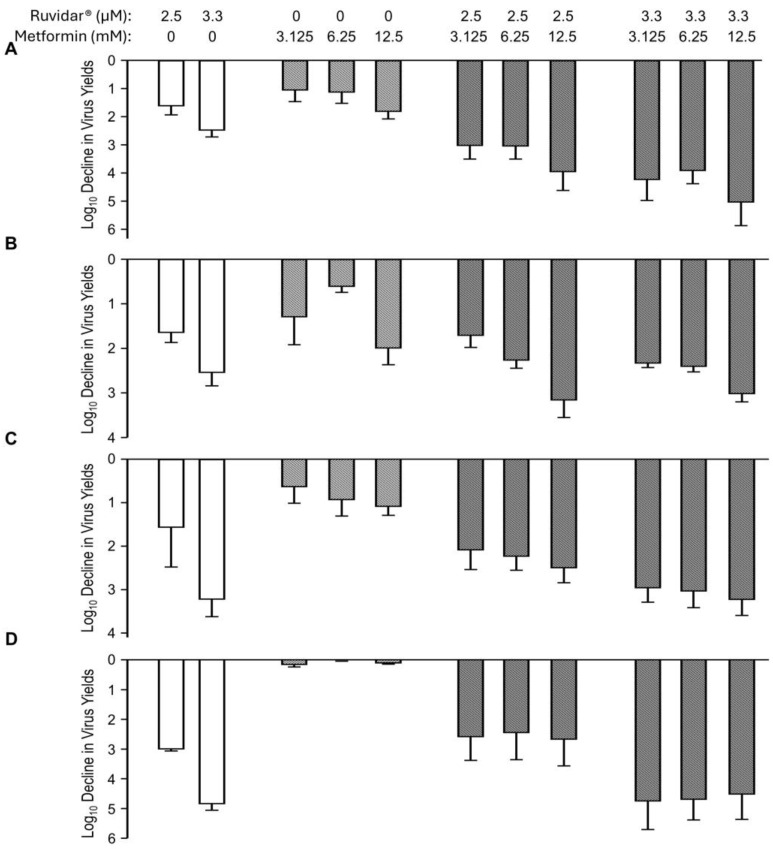
Effects of Ruvidar^®^ in combination with metformin on HSV-1 yields when added at various times pre- and post-infection. Vero cells were infected with HSV-1 at MOI ~ 1.5 and treated at 4 h pre-infection (**A**); at the same time as infection (**B**); 8 hpi (**C**); or 24 hpi (**D**) with indicated concentrations of drugs. Virus yields were determined at 68 hpi, and reductions in virus yields were compared to non-treated controls. Error bars represent SEMs from at least three replicates.

Combinations of Ruvidar^®^ and metformin are shown in Figure 5. When used alone and when added 4 h pre-infection, 2.5 and 3.3 µM Ruvidar^®^ inhibited HSV-1 replication ~1.5 and ~2.5 Log_10_, respectively (Figure 5A, left-most white bars). Similarly, when used alone and when added 4 h pre-infection, 3.125, 6.5 and 12.5 mM metformin inhibited HSV-1 replication ~1.1, ~1.2, and ~1.8 Log_10_, respectively (Figure 5A, cross-hatched bars). When used in combination, most doses had a slightly additive effect (Figure 5A, right-most light gray cross-hatched bars). The combination of Ruvidar^®^ with metformin in most cases had additive effects on HSV-1 replication inhibition when added at the same time as the infection was initiated (Figure 5B) or if 2.5 µM Ruvidar^®^ was added 8 hpi (Figure 5C); however, if 3.3 µM Ruvidar^®^ was added 8 hpi, or combinational drugs were added 24 hpi, the inhibition of HSV-1 replication was less effective than if Ruvidar^®^ was used alone (Figure 5C,D).

### 3.4. Acyclovir-Resistant HSV-1 Mutants Are Inhibited by Ruvidar^®^

**Figure 6:**
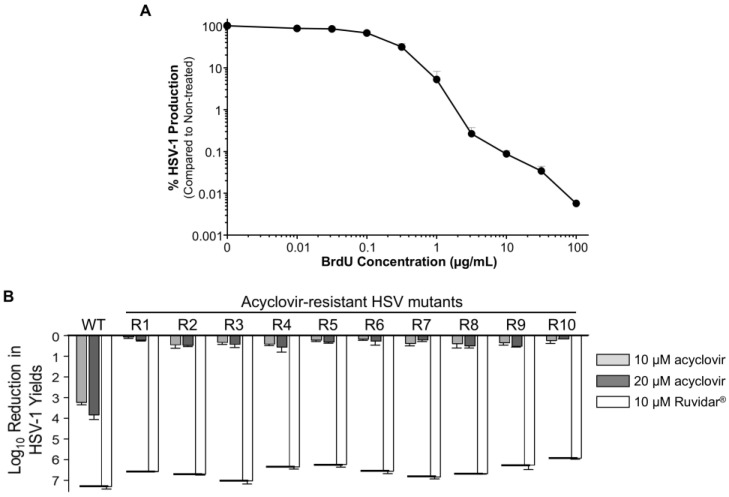
Generation of acyclovir-resistant HSV-1 (HSV^Ac-R^) mutants. (**A**) Effects of 5′-Bromo-2′-deoxyuridine (BrdU) on HSV-1 yields. Vero cells were infected with HSV-1 at MOI = 1 PFU/cell and overlayed with DMEM + 5% FBS that contained the indicated amounts of BrdU. Virus yields were determined at 48 hpi, and virus yields were compared to non-treated controls. Error bars represent S.E.M. from at least two replicates. Note that 1 μg/mL BrdU resulted in virus yields reduced to ~5% of the control. (**B**) Inhibition of wild-type (WT) HSV and HSV^Ac-R^ mutants by 10 and 20 μM acyclovir, and by 10 μM Ruvidar^™^, respectively. Vero cells were pre-treated with the indicated drugs for 2 h, infected at MOI ~ 1 PFU/cell, and overlaid with media containing same drug concentrations. Virus yields were determined at 68 hpi, and reductions in virus yields were compared to non-treated controls. Error bars represent SEMs from two replicates. The short thick horizontal bars represent the limit of detection of the plaque assays.

A major problem with HSV-1 anti-viral therapy is that acyclovir-resistant mutants can commonly develop. To test the anti-viral effects of Ruvidar^®^ on such mutants (HSV^Ac-R^), we passaged HSV-1 in the presence of increasing concentrations of acyclovir. We initially determined the concentration of 5′-Bromo-2′-deoxyuridine (BrdU) required to reduce viral progeny production 1–2 Log_10_, conditions reported to favor mutant production [18,25]. Addition of 1 μg/mL of BrdU resulted in ~5% HSV-1 production compared to that in non-drug-treated samples (Figure 6A).

We pre-treated Vero cells for 2 h with 1.25 μM acyclovir to generate HSV^Ac-R^ mutants, or with 1.25 μM Ruvidar^®^ to attempt to generate HSV^Ru-R^ mutants. We then infected the cells with HSV-1 at MOI = 1 PFU/cell, and overlaid them with DMEM that contained the same amounts of acyclovir or Ruvidar^®^, and were supplemented with 1 μg/mL of BrdU. After 48 h incubation, supernatants were used to set up 2nd passage infections with the same concentrations of acyclovir or Ruvidar^®^. The amounts of acyclovir or Ruvidar^®^ were increased through five additional passages at 3.16 and 10 μM of acyclovir or Ruvidar^®^ as detailed in the Section 2. For the 5th passage, the supernatants from the 4th passage were titrated under agarose that contained 3.16 or 10 μM acyclovir or Ruvidar^®^, respectively. Ten well-separated plaques were picked from the acyclovir-treated plates into media containing 10 μM acyclovir and passaged in the presence of 10 μM drug for 2 additional passages. We were unable to recover any plaques from the Ruvidar^®^-treated plates, so attempts to generate HSV^Ru-R^ mutants were discontinued.

These putative HSV^Ac-R^ mutants, and wild-type HSV-1, were then used to infect Vero cells that had been pre-treated for 2 h with either 0, 10, or 20 μM acyclovir or 10 μM Ruvidar^®^. Cells were infected with HSV-1 at MOI ~ 1.5, overlaid with 0, 10, or 20 μM acyclovir or 10 μM Ruvidar^®^, respectively, and virus yields were determined at 68 hpi (Figure 6B).

Wild-type (wt) HSV-1 grew to about 1 × 10^8^ PFU/mL, and growth was inhibited 3.26 Log_10_ (>1850-fold) by 10 μM acyclovir, 3.86 Log_10_ (>8000-fold) by 20 μM acyclovir, and 7.34 Log_10_ (>22,000,000-fold) by 10 μM Ruvidar^®^, comparable to earlier results. The various HSV^Ac-R^ mutants grew to between 4.1 × 10^6^ to 8 × 10^7^ PFU/mL in the absence of drugs and were inhibited no more than 3.2-fold by 10 μM acyclovir and no more than 4.4-fold by 20 μM acyclovir, but were inhibited >890,000-fold by 10 μM Ruvidar^™^ (Figure 6B).

### 3.5. Ruvidar^®^ Also Inhibits HSV-1 and Acyclovir-Resistant HSV-1 Mutants in U251 Astrocytes

**Figure 7:**
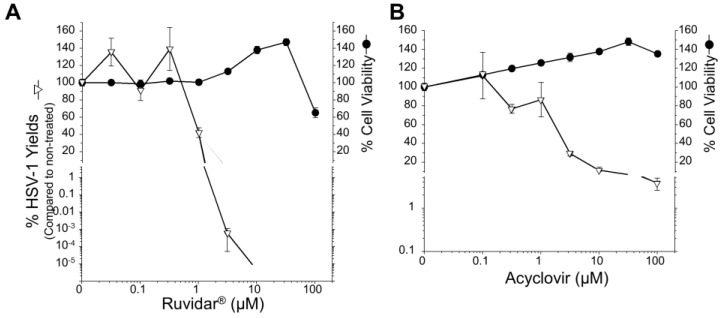

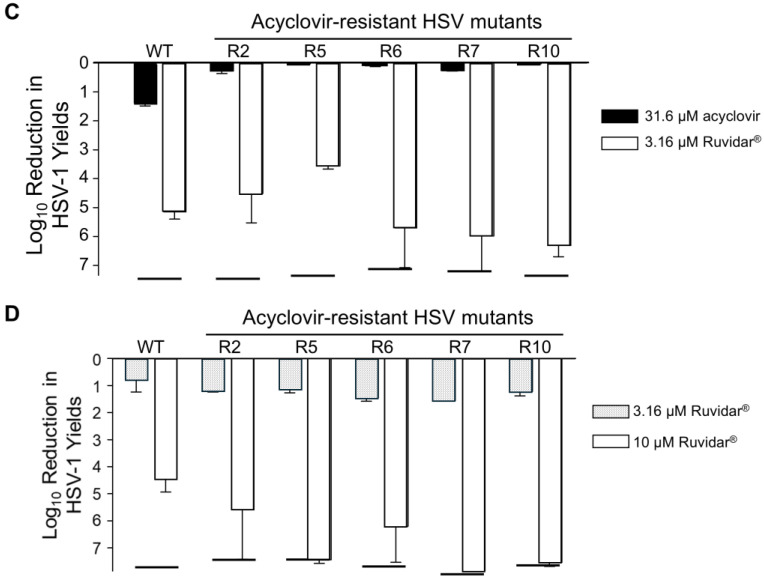
Effects of Ruvidar^®^ and acyclovir on wild-type (WT) HSV-1 and acyclovir-resistant HSV-1 (HSV^Ac-R^) yields in human U251 glioblastoma astrocytoma cells. Effects of Ruvidar^®^ (**A**) and acyclovir (**B**) on U251 cell viability and HSV-1 yields. U251 cells were treated with the indicated concentrations of drugs for 72 h to measure cell viability, or cells were pre-treated for 4 h with the indicated drug concentrations, infected with HSV-1 at MOI ~ 0.01, incubated in the presence of same drug concentrations as pre-treatment for 68 h, and virus yields were determined. Error bars represent SEMs from at least three replicates. The broken line at bottom of the virus yield curve in A denotes reaching the limit of detection. (**C**) Prophylactic effects of acyclovir and Ruvidar^®^ on HSV-1 and HSV^Ac-R^ yields when drugs were added pre-infection. U251 cells were pre-treated for 2 h with indicated drugs, infected with HSV-1 or each of five HSV^Ac-R^ clones at MOI ~ 1.5, and incubated in the continued presence of appropriate drugs. Virus yields were determined at 68 hpi, and reductions in virus yields were compared to non-treated controls. Error bars represent SEMs from two replicates. (**D**) Therapeutic effects of Ruvidar^®^ on HSV-1 and HSV^Ac-R^ yields when drugs were added 24 h post-infection. U251 cells were infected with HSV-1 or each of the five HSV^Ac-R^ clones at MOI ~ 1.5, treated at 24 hpi with the indicated drugs, and incubated in the continued presence of appropriate drugs. Virus yields were determined at 68 hpi, and reductions in virus yields were compared to non-treated controls. Error bars represent SEMs from two replicates. The short thick horizontal bars represent the limit of detection of the plaque assays.

All previous work had been performed in monkey kidney Vero cells. Astrocytes are another cell type that can be infected by several herpesviruses, including HSV-1 [26,27]. To test whether these drugs could also inhibit HSV-1 in this more relevant cell type, we initially pre-treated human U251 glioblastoma astrocytoma cells with different concentrations of acyclovir or Ruvidar^®^ and determined cell viabilities and the capacity of WT HSV-1 to replicate (Figure 7). Cell viability remained >50% at all tested Ruvidar^®^ concentrations, and wild-type HSV-1 yields were reduced >10,000-fold by 3.16 μM Ruvidar^®^ (Figure 7A). Cell viability also was unaffected by any tested acyclovir concentration up to 100 μM and HSV-1 yields were reduced ~10-fold at 10 μM acyclovir and ~25-fold at 100 μM (Figure 7B).

Comparison of the dose response curves of the two drugs suggests that higher doses of acyclovir and lower doses of Ruvidar^®^ are needed in the U251 cells compared to the Vero cells to inhibit HSV-1 to the same extent (compare Figure 7A to Figure 1, left, and Figure 7B to Figure 1, center). For example, 10 μM acyclovir induced nearly a 1000-fold reduction in HSV yields in Vero cells but only about a 10-fold reduction in U251 cells (*p* < 10^−5^ by *T*-test), whereas 3.16 μM Ruvidar^®^ induced about a 1000-fold reduction in HSV yields in Vero cells but more than a 100,000-fold reduction in U251 cells (*p* = 0.003 by *T*-test).

We then pre-treated U251 cells with nothing, 31.6 μM acyclovir, or 3.16 μM Ruvidar^®^. Cells were infected with wt HSV-1, or with five selected HSV^Ac-R^ mutants at MOI ~ 1.5, overlaid with media lacking drugs, or media containing the same drugs, and virus yields were determined at 68 hpi (Figure 7C). WT HSV-1 grew to ~2 × 10^8^ PFU/mL yield in the absence of drugs. Yields were inhibited 25-fold by 31.6 μM acyclovir, and >165,000-fold by 3.16 μM Ruvidar^®^. All of the acyclovir-resistant clones grew to between 5.95 × 10^7^ and 2.2 × 10^8^ in the absence of drugs, showed <2-fold reduction for 31.6 μM acyclovir, and, except in the case of HSV^Ac-R5^, which was inhibited 3783-fold, they showed a > 170,000-fold reduction to 3.16 μM Ruvidar^®^, indicating they all remained resistant to acyclovir but remained sensitive to Ruvidar^®^ in this other cell system.

To test the therapeutic efficacy of Ruvidar^®^ on already-established infections of wt HSV-1 and of the HSV^Ac-R^ mutants, we also infected U251 cells with the same six virus clones (wt + five HSV^Ac-R^ mutants) at MOI ~ 1.5 PFU/cell, incubated the infections for 24 h, then added no drug, or 3.16 or 10 μM of Ruvidar^®^. Infections were harvested 44 h later and virus yields were determined to see if therapeutic application of Ruvidar^®^ would affect HSV^Ac-R^ replication (Figure 7D).

WT HSV-1 grew to almost 3 × 10^8^ PFU/mL, and growth was inhibited 10-fold by 3.16 μM Ruvidar^®^ and 47,000-fold by 10 μM Ruvidar^®^, respectively, even when added 24 h after infection had started. The various HSV^Ac-R^ mutants grew to ~1 × 10^8^ PFU/mL in U251 cells in the absence of drugs, were inhibited >14-fold by 3.16 μM Ruvidar^®^, and were inhibited >13,000,000-fold by 10 μM Ruvidar^®^, indicating that therapeutic application of Ruvidar^®^ remained effective, even against acyclovir-resistant HSV-1.

### 3.6. Ruvidar^®^ Protects HSV-1-Infected Cells from Cytopathology

**Figure 8:**
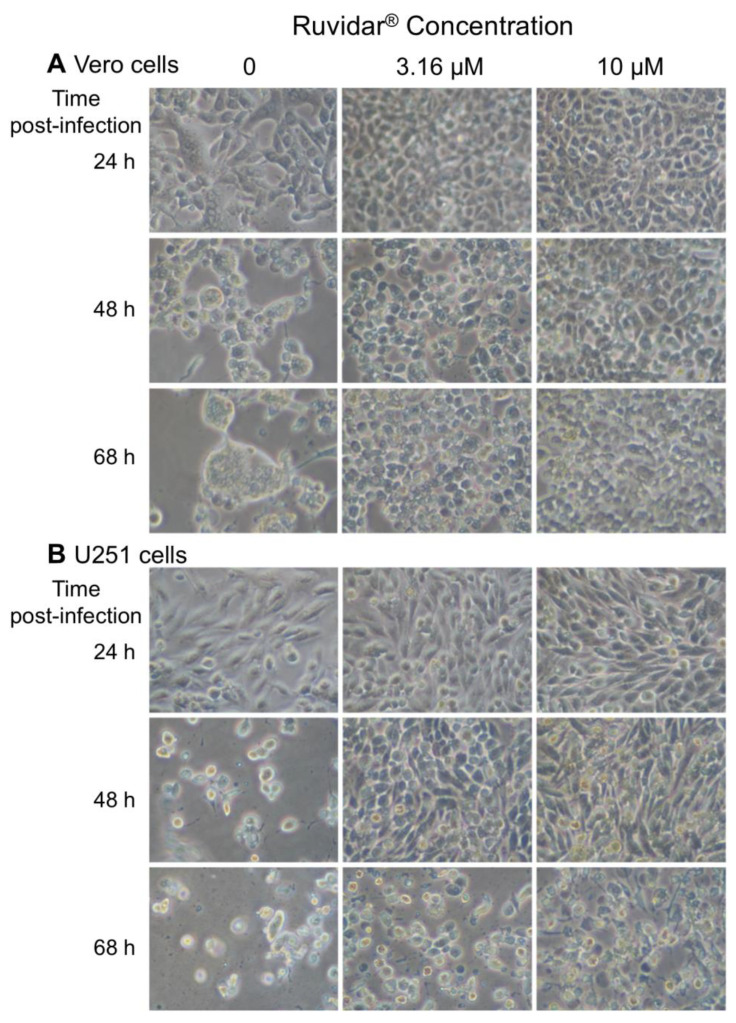
Cytopathic effects induced in (**A**) Vero and (**B**) U251 cells in the presence of 0, 3.16, and 10 μM Ruvidar^®^ at the indicated times post-infection (left). Cells were pre-treated with the indicated concentrations of compound for 2 h, infected with HSV-1 at MOI ~ 1.5, and incubated in the same concentrations of compounds.

To determine if Ruvidar^®^ application would affect the HSV-1-induced cytopathic effect (CPE), we pre-treated both types of cells with 0, 3.16, or 10 μM Ruvidar^®^, infected with HSV-1 at MOI 1.5, and monitored cell morphology over time (Figure 8). HSV-1 infection induced a small amount of CPE in non-drug-treated cells by 24 hpi in both cell types, and there was substantial CPE in both non-drug-treated cells by 48 hpi. By contrast, there was little, if any, CPE in both cell types treated with either drug concentration by 48 hpi. There also was little, if any, CPE in the Vero cells treated with either drug concentration by 68 hpi. U251 cells showed stress in both drug concentrations by 68 hpi.

## 4. Discussion

The herpesviruses are significant human pathogens. Although many, such as Herpes Simplex Virus (HSV), rarely cause mortality, they are ubiquitous and virtually the entire human population has been exposed to one or another of the eight currently recognized human herpesvirus types. These viruses are capable of entering human hosts by various methods and can damage numerous tissues, including the central nervous system (CNS), which can be fatal (reviewed in [6]).

Furthermore, in addition to causing acute infections, these agents can establish life-long latency and spontaneously reactivate. For example, the disease Shingles occurs when Varicella Zoster virus, the causative agent of Chickenpox, is reactivated.

There are several treatments for HSV. Vaccines have been developed against a few herpesviruses, such as Varicella Zoster [28], but despite decades of research, such vaccines are not yet available for HSV-1 [29]. To date, anti-HSV-1 strategies have focused on pharmacologic therapies; including acyclovir [19,20,21], its derivatives such as famciclovir and valaciclovir [9,30], and other compounds, such as foscarnet [10,31] and cidofovir [10,32]. Viruses mutate and therefore can develop resistance to anti-viral treatments [3,4,5]; thus, there is need to develop additional strategies.

Activated small molecules are a potential strategy that is effective against various cancers [33,34,35,36,37], bacterial pathogens [38,39], and viruses [40,41,42,43,44]. This methodology is based upon the use of a small molecule that is administered to the pathogen in question, then activated by exposure to a particular form of energy—in this case, wavelength(s) of light—to produce Reactive Oxygen Species (ROS), specifically singlet oxygen, which causes microbial or cancer oxidative stress and subsequent death [39,42]. Originally found to kill paramecia, a variety of different light-activated small molecules have been developed and tested over the years, with third- and fourth-generation light-activated small molecules now under investigation (reviewed in [36,45]).

Our prior work showed that Ruvidar^®^ could effectively inactivate numerous human viruses when added to a virus at nanomolar concentrations [11], and that, while not quite as effective at equivalent doses, Ruvidar^®^ could inactivate all tested viruses when not activated by light. Thus, Ruvidar^®^ could potentially be used in clinical niches, where light is unable to penetrate or be activated by other methodologies, such as ionizing radiation or other drugs, dependent on the application. This latest research has determined what effects, if any, Ruvidar^®^ could have on the replication of HSV-1 in model cell culture systems of Vero and U251 astrocytoma cells; whether Ruvidar^®^ was as effective or more effective than acyclovir, the current “Gold Standard”; and whether combinational treatments could have additive anti-HSV-1 effects.

When used prophylactically (added prior to infection), acyclovir is an effective anti-herpetic agent (as previously demonstrated (i.e., [19,20,21]), with 10 μM inhibiting HSV-1 yields almost 99.9% in Vero cells. Acyclovir was not as effective in U251 cells, with 10 μM inhibiting HSV-1 yields only by about 10%.

Ruvidar^®^ was significantly more effective than acyclovir, especially in the astrocytoma cells, with 3.16 μM inhibiting HSV-1 yields > 99.9% and 10 μM inhibiting HSV-1 yields almost 10-fold more in Vero cells, and 3.16 μM inhibiting HSV-1 yields > 5 Log_10_ in the U251 cells. Metformin also showed anti-HSV-1 activity when added prior to infection, with ~20 mM inhibiting HSV-1 approximately 99.9% and 25 mM inhibiting HSV-1 almost 10-fold more in Vero cells. Thus, of these three tested agents, Ruvidar^®^ was by far the most effective on a molar basis. The differences in the effectiveness of acyclovir and Ruvidar^®^ in the different cell types indicate that in addition to its virucidal activity [11], there are cell-specific factor(s) that contribute to Ruvidar^®^’s anti-HSV activity.

When used therapeutically, Ruvidar^®^ was substantially more effective than the other two compounds due to the fact that no tested acyclovir or metformin concentrations were effective if added to Vero cells 24 hpi. On the contrary, Ruvidar^®^ remained highly effective, with 5 μM inhibiting HSV-1 yield by approximately 99%, while 10 and 20 μM inhibited HSV-1 yields by approximately 5 Log_10_ and 7 Log_10_, respectively, in Vero cells. Ruvidar^®^ also remained highly effective when used therapeutically in U251 cells, with 10 μM inhibiting yields of wt and all tested HSV^Ac-R^ mutants more than 4 Log_10_. This suggests that Ruvidar^®^ could be used effectively both before and during HSV infection, making it a much more attractive and commercially viable anti-viral therapeutic against this ubiquitous virus.

Viruses are more susceptible to mutation than eukaryotic cells and therefore can escape anti-viral treatment. This has been seen in many cases, particularly with RNA viruses [3,4,5], but also with DNA viruses. For example, several clinical HSV isolates have become resistant to acyclovir [46], making it necessary to switch to derivatives such as ganciclovir [8,10] and valacyclovir [10,46], or to other compounds, such as cidofovir or foscarnet [10,32]. When Ruvidar^®^ was evaluated against a number of acyclovir-resistant HSV-1 mutants (HSV^Ac-R^), it confirmed that they were resistant to acyclovir in both U251 and Vero cells and that Ruvidar^®^ remained effective against all tested HSV^Ac-R^ in both cell types.

Using combinational drug therapy leads to reduced, or slower, resistance development, particularly if the drugs target different aspects of virus replication [22,23,24,47]. To ascertain whether the same held true for our tested compounds, we tested whether Ruvidar^®^, in combination with either metformin, which activates Ruvidar^®^ (https://theralase.com/theralase-demonstrates-unique-ability-to-activate-rutherrin-with-diabetes-drug/, accessed on 21 February 2025), or acyclovir, could increase the anti-HSV-1 activity compared to any drug used alone. Combinational therapy had an additive effect in the majority of cases. For example, 2.5 μM Ruvidar^®^ alone inhibited HSV-1 yields 41-fold, and 2.5 μM acyclovir alone inhibited HSV-1 yields 75-fold, but the combination of 2.5 μM of each inhibited HSV-1 yields > 14,000-fold when used prophylactically. There also was an additive effect when these doses were added 8 hpi. The combination of 3.3 μM Ruvidar^®^ with 10 μM acyclovir appeared to be synergistic when used prophylactically. When added prior to infection, 3.3 μM Ruvidar^®^ inhibited HSV-1 yields 298-fold and 10 μM acyclovir inhibited HSV-1 yields 1892-fold. Synergistically, the combination inhibited HSV-1 yields > 31 million-fold (*p* = 0.015). Synergistic and additive effects were also seen when Ruvidar^®^ and metformin were used in combination, although some combinations, particularly when tested therapeutically, were not always additive, and in some cases they were subtractive in nature. For example, 3.3 μM Ruvidar^®^ alone added at the time of infection inhibited HSV-1 replication 346-fold (2.539 Log_10_), 3.125 mM metformin alone inhibited HSV-1 replication 19-fold (1.29Log_10_), but the combination, added at the time of infection, only inhibited HSV-1 replication 215-fold (2.33 Log_10_) instead of nearly 6734-fold (3.828Log_10_). The authors attribute this unexpected subtractive/nil effect to the timing of when the various agents were added to virus-infected cells versus the agent’s various mechanisms of action against the virus. In other words, the proliferation stage of the virus versus when the agent was administered plays a key role in the synergistic/additive effect of the agents.

In addition to greatly reducing HSV-1 replication, whether used prophylactically or therapeutically, Ruvidar^®^ treatment also reduced and/or delayed cytopathology (CPE) in both tested cell types, with the CPE reduction being more apparent in Vero cells than in U251 cells. Thus, Ruvidar^®^ appears to be a highly effective anti-HSV-1 agent, as demonstrated by the detailed experiments completed. The next steps include the completion of a Phase I/II clinical study in an attempt to replicate these results in a clinical model.

## 5. Conclusions

In conclusion, the above results indicate that Ruvidar^®^ is a much more effective anti-HSV treatment than either acyclovir or metformin. These studies involved treating a population of cells either before they were exposed to HSV-1 (prophylactically), in which viral replication was dramatically reduced by all tested pharmacologic compounds, or after virtually every cell had been infected (therapeutically). While acyclovir and metformin had little, if any, beneficial effect once infection was established, Ruvidar^®^ continued to have a dramatic inhibitory effect. In a normal human infection setting, a small proportion of cells are initially infected and subsequent progeny viruses infect a greater number of additional cells. Thus, adding acyclovir or metformin alone after symptoms present has no beneficial effect upon the already-infected cells; however, treatment with any of the compounds could help reduce viral replication once neighboring non-infected cells become infected. This inhibitory effect was significantly enhanced when Ruvidar^®^ was used in combination with acyclovir or metformin. Ruvidar^®^ is also highly effective at protecting cells from ongoing HSV infection and highly effective against acyclovir-resistant HSV-1 mutants.

## Data Availability

All data are contained within the manuscript figures.

## Abbreviations

The following abbreviations are used in this manuscript:

DMEM	Dulbecco’s Minimal Essential Medium
DMSO	Dimethyl Sulfoxide
HSV	Herpes Simplex Virus
